# Endocardial left ventricular pacing

**DOI:** 10.1007/s00059-021-05074-7

**Published:** 2021-10-25

**Authors:** Mark K. Elliott, Vishal S. Mehta, Baldeep Singh Sidhu, Steven Niederer, Christopher A. Rinaldi

**Affiliations:** 1 School of Biomedical Engineering and Imaging Sciences, St Thomas’ Hospital, King’s College London, SE1 7EH London, UK; 2 Department of Cardiology, Guy’s and St Thomas’ NHS Foundation Trust, London, UK

**Keywords:** Cardiac resynchronization therapy, Hemodynamics, Heart failure, Leadless pacing, Conduction system pacing, Kardiale Resynchronisationstherapie, Hämodynamik, Herzinsuffizienz, Kabellose Stimulation, Stimulation des Reizleitungssystems

## Abstract

Cardiac resynchronization therapy (CRT) is an effective treatment for dyssynchronous heart failure; however, 30–50% of patients fail to improve after implant. Endocardial left ventricular (LV) pacing is an alternative therapy for patients who do not respond to conventional CRT or in whom placement of a lead via the coronary sinus is not possible. It enables pacing at a wide variety of sites, without restrictions due to coronary sinus anatomy, and there is evidence of superior electrical resynchronization and hemodynamic response compared with conventional epicardial CRT. In this article, we discuss the potential advantages and disadvantages of endocardial LV pacing compared with conventional CRT, review the evidence for the delivery of endocardial LV pacing using both lead-based and leadless systems, and explore possible future directions of this novel technology.

## Abstract (de)

Die kardiale Resynchronisationstherapie (CRT) stellt eine effektive Behandlung der dyssynchronen Herzinsuffizienz dar, jedoch kommt es bei 30–50 % der Patienten nach Implantation nicht zu einer signifikanten klinischen Verbesserung. Die endokardiale linksventrikuläre (LV-)Stimulation bietet eine alternative Therapie für Patienten, deren Erkrankung sich unter konventioneller CRT nicht bessert oder bei denen die Implantation einer Elektrode über den Koronarsinus nicht möglich ist. Sie ermöglicht eine Stimulation von einer Vielzahl linksventrikulärer Positionen aus ohne die Limitationen der Koronarsinusanatomie, und es bestehen Belege für eine überlegene elektrische Resynchronisation und Hämodynamik durch endokardiale verglichen mit konventioneller epikardialer CRT. In dieser Übersicht diskutieren die Autoren die möglichen Vor- und Nachteile einer endokardialen LV-Stimulation im Vergleich zur konventionellen CRT, fassen die Evidenz für die endokardiale LV-Stimulation sowohl für elektrodenbasierte wie auch für kabellose Systeme zusammen und untersuchen mögliche zukünftige Entwicklungen dieser neuen Technologie.

Cardiac resynchronization therapy (CRT) is an effective treatment that improves symptoms and mortality in patients with heart failure and electrical dyssynchrony [1]. Conventional CRT is delivered using endocardial leads in the right atrium and right ventricle and an epicardial left ventricular (LV) lead that is placed in a branch of the coronary sinus, thus enabling both atrioventricular and ventriculoventricular resynchronization. However, between 30 and 50% of patients do not respond after implantation [2]. Nonresponse to CRT is multifactorial, involving poor patient selection, suboptimal left ventricular lead position, ineffective CRT delivery, and suboptimal optimization of device programming [2]. In addition, conventional CRT cannot be achieved in 8–10% of patients due to venous occlusion, nonviable coronary sinus anatomy, myocardial scar, or phrenic nerve stimulation [3, 4]. While the use of quadripolar leads has helped overcome some of these issues and improve outcomes [5], in a significant subgroup of patients optimal CRT with an LV lead in a branch of the coronary sinus cannot be achieved. Endocardial pacing enables stimulation of the LV endocardium at any location, unrestricted by coronary venous anatomy, therefore enabling pacing at the latest activation site and away from myocardial scar. It can be delivered using a conventional pacing lead, usually implanted via a transseptal interatrial approach, or via the leadless WiSE-CRT system (EBR systems, Sunnyvale, CA, USA).

In this review, we outline the potential advantages and disadvantages of endocardial LV pacing compared to conventional CRT and discuss the evidence for both lead-based and leadless endocardial pacing in clinical practice.

## Potential advantages of left ventricular endocardial pacing

### Optimizing site of left ventricular pacing

Optimal LV lead positioning within the coronary sinus tributaries is an important determinant of CRT response. Randomized trials have demonstrated superior CRT response and lower mortality and hospitalizations for heart failure when an echo-guided approach is used to target the LV lead to the site of the latest mechanical activation [6, 7]. Small pilot studies of magnetic resonance imaging-guided and computed tomography-guided LV lead placement have demonstrated the additional benefits of avoiding areas of myocardial scar [8, 9]. More recently, the international multicenter RADI-CRT trial demonstrated superior LV remodeling when a pressure wire was used to choose the optimal coronary sinus branch based on acute hemodynamic response [10]. While these studies demonstrate that targeting the LV lead to the optimal site is superior to empirical LV lead placement, most patients have a limited number of coronary sinus branches available for placement of a lead. Endocardial LV pacing has the advantage of facilitating stimulation of the LV at any anatomical location, thus increasing the chance of pacing at the optimal site. Several human mechanistic studies have compared endocardial LV pacing at multiple locations with conventional pacing from a coronary sinus lead [11–14]. In these studies, the optimal location for LV pacing, determined by acute hemodynamic response, varied greatly between patients, but was superior for the optimal endocardial site compared to epicardial pacing from the coronary sinus. This highlights the importance of an individualized approach to LV lead placement, and that endocardial pacing enables the optimal location to be targeted.

### Hemodynamic and electrical resynchronization benefits

The aforementioned initial mechanistic studies did not demonstrate superior hemodynamic performance when pacing the same site endocardially versus epicardially [11–14]. However, in these pacing protocols, a limited number of epicardial locations (often a single site) were tested. In a subsequent study by Behar et al., eight patients with ischemic cardiomyopathy and existing CRT systems underwent temporary epicardial and endocardial pacing [15]. In contrast to previous studies, multiple epicardial and endocardial pacing locations were tested. Superior acute hemodynamic response and electrical resynchronization (on surface ECG) were demonstrated when pacing the same location endocardially versus epicardially. The discrepancy in the findings may be due to the fact that the previous studies did not test epicardial pacing in the optimal location. Animal studies support the findings that endocardial pacing is superior to epicardial pacing in the same location. In a canine model of acute left bundle branch block (LBBB) induced by radiofrequency ablation, hemodynamic assessment and electrical mapping were performed during epicardial and endocardial LV pacing [16]. Epicardial pacing was performed via two multi-electrode bands positioned around the epicardium of the heart. This allowed epicardial pacing to be carried out in multiple sites, unrestricted by coronary sinus anatomy. Benefits in acute hemodynamics and LV activation times were significantly greater during biventricular endocardial pacing compared to biventricular epicardial pacing at the same site. In a subsequent study from the same group, similar hemodynamic and electrical benefits of biventricular endocardial pacing were demonstrated in canine models of myocardial infarction with LBBB and chronic LBBB with heart failure [17].

These studies suggest that the hemodynamic and electrical resynchronization benefits seen with endocardial pacing are due to more than simply accessing the optimal pacing location within the LV. It has been theorized that the superior acute hemodynamic response observed during endocardial pacing is explained by more rapid LV activation, which in turn is due to accessing fast-conducting tissue within the endocardium, or retrograde conduction in the distal Purkinje network. This idea is supported by a computational electrophysiology simulation study where the addition of fast-conducting endocardial tissue to both canine and human heart models explained the faster activation times observed during LV endocardial pacing compared to epicardial pacing [18].

### Repolarization benefits

Another potential benefit of endocardial LV pacing is a reduction in dispersion of repolarization, which in turn may reduce the risk for arrhythmogenesis. Epicardial LV pacing reverses the physiological pattern of activation and repolarization within the myocardial wall, and has been demonstrated to increase the QT interval and transmural dispersion of repolarization in animal studies [19, 20]. Increased local dispersion of repolarization has also been demonstrated during epicardial pacing in close proximity to scar in computational modeling studies [21, 22]. However, while CRT-induced ventricular tachycardia has been reported [23], conventional CRT appears to have a significantly beneficial effect on the risk of ventricular arrhythmia, most likely due to the associated reverse LV remodeling. In a recently published substudy of the MADIT-CRT trial, patients with CRT-defibrillators had a significantly lower rate of ventricular arrhythmia in the follow-up period compared to patients with implantable cardioverter-defibrillators (ICD) alone [24], and CRT responders have been shown to have lower rates of ventricular arrhythmia compared to non-responders in a meta-analysis [25].

Endocardial LV pacing may restore the physiological transmural pattern of activation and repolarization. In a canine LBBB model study, biventricular epicardial pacing, but not endocardial pacing, created a significant transmural dispersion of repolarization [16]. This is supported by a computational modeling study where the high repolarization gradients observed during epicardial pacing in close proximity to scar were not found during endocardial pacing [21]. How the observed effects on repolarization translate into risk of ventricular arrhythmia in clinical practice remains unclear; however, they suggest that endocardial LV pacing may be less arrhythmogenic than epicardial pacing, particularly in patients with ischemic cardiomyopathy.

## Lead-based left ventricular endocardial pacing

The delivery of endocardial LV pacing in clinical practice is largely restricted to patients in whom conventional CRT has failed or is not feasible, and randomized trials comparing endocardial pacing with conventional CRT are lacking. There is currently no dedicated delivery system for implanting pacing leads into the LV endocardium, and a variety of techniques have been described. The largest observational study of endocardial LV pacing is the prospective multicenter ALSYNC trial, in which 132 patients who had failed or were unsuitable for conventional CRT underwent endocardial LV pacing using a transseptal interatrial approach via standard subclavian venous access [26]. Implantation was successful in 89% of patients, although the lead could be fixated in the desired location in only 81% of cases, thus highlighting the technical challenges of LV endocardial lead placement. Five cases of postoperative stroke and 14 TIA episodes were reported, and all patients required long-term anticoagulation with warfarin. At 6 months, 55% of patients demonstrated significant LV reverse remodeling (reduction in LV end-systolic volume [LVESV] ≥ 15%) and 59% of patients reported improvement by at least one New York Heart Association (NYHA) functional class. Performance cannot be directly compared against conventional CRT, as the study did not have a control group and the patient cohort significantly differed from those undergoing standard de novo CRT devices, with 55% having a previous failed attempt at CRT implantation, 22% having suboptimal coronary sinus anatomy, and 23% being defined as CRT nonresponders. Interestingly, of the patients defined as CRT nonresponders, 47% demonstrated significant reverse LV remodeling after endocardial CRT.

Other evidence for lead-based endocardial LV pacing is largely restricted to small single-center case series, and is summarized in two recent meta-analyses that included 362 and 384 patients, respectively [27, 28]. In addition to the transseptal interatrial approach used in the ALSYNC trial, transseptal interventricular and transapical approaches to the LV have also been reported. While the quality of the evidence in these meta-analyses was limited, the procedural success was high, with an estimated overall rate of symptomatic improvement reported to be 82% [28]. The main concern around delivery of lead-based endocardial LV pacing remains the risk of thromboembolic complications and the need for long-term anticoagulation. The overall stroke rate reported in one meta-analysis was 3.3–4.2 per 100 patient years, which is significantly higher than reported rates in equivalent heart failure trial populations [27]. Other concerns include the risk of impairment of and adhesion to the mitral valve by the transseptal lead and the risk associated with infected leads, as left-sided vegetations can lead to systemic embolic complications, and extraction of endocardial LV leads may be more complicated than right-sided leads. The reported rate of lead infection in one meta-analysis was 3.6% (2 per 100 patient years), which is higher than for conventional CRT [27]. While there were no complications associated with the two leads that were extracted in the included studies, the risks associated with extraction of leads that have been in situ for extended periods of time are unknown. It is also important to note that the majority of patients included in the meta-analyses were in case reports or case series, thus raising the possibility of under-reporting of complications due to publication bias.

More recently, a multicenter observational study has reported longer-term outcomes of 88 patients who underwent endocardial LV pacing using the Jurdham procedure [29]. This technique uses a transseptal intra-atrial approach for endocardial lead placement in the lateral LV wall via femoral venous access, followed by percutaneous snaring of the proximal lead tip via the subclavian vein to facilitate connection to the CRT generator. Patients were included if they had a failed attempt at coronary sinus lead placement or were nonresponders to conventional CRT. In addition, some patients indicated for CRT who were already taking long-term oral anticoagulation were offered endocardial LV pacing as first-line therapy. The procedure was successful in all cases, and patients were followed up for a mean of 32.88 ± 61.52 months, which is longer than previous studies. A remarkably high response rate was reported, with all patients having improvement in ≥ 1 NYHA class and LV ejection fraction improving by 10–20 percentage points in 11% of patients and > 20 percentage points in 82% of cases. However, the rate of thromboembolic complications during follow-up was 10.2% (TIA: 1.52 per 100 patient years; stroke: 3.06 per 100 patient years).

## Wireless left ventricular endocardial pacing

**Fig. 1:**
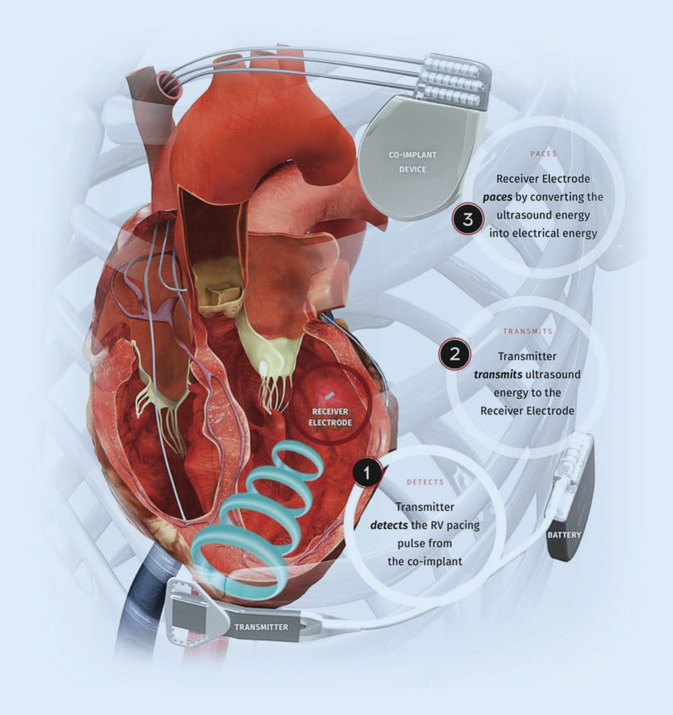
Components of the WiSE-CRT system. (Reproduced with permission from EBR Systems)

Left ventricular endocardial pacing can also be delivered wirelessly using the WiSE-CRT system. The components of the WiSE-CRT system are demonstrated in Fig. 1. It consists of a transmitter that is implanted over the intercostal muscle in a pre-identified intercostal space and connected to a generator, which is placed in the adjacent mid-axillary line. The wireless endocardial electrode is implanted via a retrograde aortic approach using femoral arterial access, or via an interatrial transseptal approach using femoral venous access. The system requires the presence of a co-implant capable of delivering continuous right ventricular (RV) pacing. After sensing the RV pacing signal from the co-implant, the transmitter delivers a focused beam of ultrasound energy to the endocardial electrode, which converts this into electrical energy to pace the LV myocardium and achieve near-simultaneous biventricular pacing. This system has several potential advantages over lead-based LV endocardial pacing. The endocardial electrode becomes fully endothelialized, which may reduce the long-term risk of thromboembolic complications, and negates the need for long-term anticoagulation. Furthermore, the significant risks associated with extraction of longstanding leads, due to infection or lead failure, can be avoided.

The feasibility of the system was initially reported in the WiSE-CRT study, in which 17 patients who had a previous failed attempt at CRT implant, were CRT nonresponders, or were indicated for CRT upgrade, underwent WiSE-CRT implantation [30]. However, the trial was terminated early due to three procedure-related pericardial effusions, and resulted in a re-design of the delivery sheath to incorporate a balloon at the distal tip, thus reducing the risk of trauma to the LV wall. The subsequent multicenter SELECT-LV trial, using the re-designed system, reported outcomes of 35 attempted WiSE-CRT implants [31]. Procedural success was high (97.1%), with 33 of 34 patients meeting the primary efficacy endpoint of successful biventricular pacing at 1 month. At 6 months, 84.8% of patients had an improvement in clinical composite score, and 66% showed echocardiographic response (defined as absolute improvement in LV ejection fraction ≥ 5%). While there were no periprocedural pericardial effusions, complication rates remained relatively high (8.6% at 24 h and 22.9% at 1 month). One procedure-related death was reported, due to fatal ventricular arrhythmia during implantation, there was one embolization of the endocardial electrode (without complication), and one patient required surgical repair of a femoral artery fistula. During follow-up, one patient with underlying atrial fibrillation had a stroke, although this was likely related to sub-therapeutic anticoagulation at the time.

The largest report on WiSE-CRT implantation in clinical practice to date is a multicenter international registry of 90 patients from 14 European centers [32]. Procedural success was again high, with chronic delivery of biventricular pacing achieved in 94.4% of patients and 69.8% of patients reporting an improved clinical composite score at 6 months. In the subgroup of patients in whom echocardiography data were available, 58.1% demonstrated significant LV remodeling (reduction in LVESV ≥ 15%). Reported rates of acute (< 24 h), intermediate (24 h to 1 month), and chronic (1–6 months) were 4.4%, 18.8%, and 6.7% respectively. This included three procedure- or device-related deaths, two of which were secondary to LV perforation. Only one stroke was reported in the follow-up period, which was not thought to be device-related. Of note, 76% of the complications occurred within a center’s first ten implants, suggesting a significant initial learning curve when implanting this system. In a subanalysis of 20 nonresponders to conventional CRT who underwent WiSE-CRT implantation, 55.6% of patients demonstrated improvement in their clinical composite score and 66.7% had an echocardiographic response (either a reduction in LVESV ≥ 15% or improvement in LVEF ≥ 5%) at 6 months [33]. This demonstrates the utility and clinical efficacy of the WiSE-CRT system in patients who do not respond to conventional CRT, and supports previous evidence from mechanistic studies that endocardial LV pacing may be superior to conventional epicardial pacing via the coronary sinus.

## Future directions with WiSE-CRT

### SOLVE-CRT

An international, randomized, sham-controlled trial of the WiSE-CRT system (SOLVE-CRT) is currently enrolling participants. The initial aim of the study was to recruit 350 patients who had either failed to respond to, or were unable to receive, conventional CRT [34]. After implantation, patients would be randomized 1:1 to the device turned ON or OFF, with follow-up at 6 months. Due to the impact of the COVID-19 pandemic on enrolment, the trial is continuing with a modified protocol, with all patients being recruited to a single-arm treatment-only phase, and excluding patients who have already received, but failed to respond to, conventional CRT [35]. This is the largest and first randomized study of endocardial LV pacing, and will provide important insights into the safety and efficacy of this novel technology.

### Completely leadless CRT

**Fig. 2:**
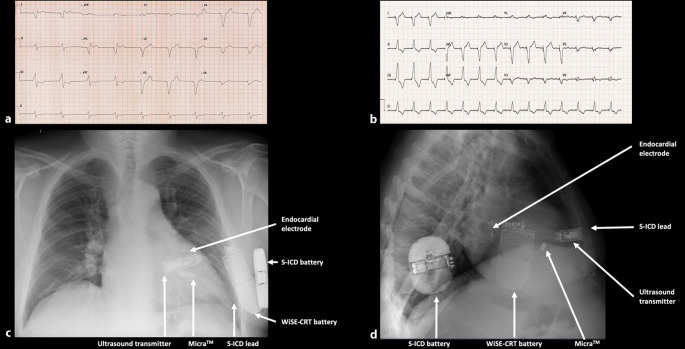
Completely leadless cardiac resynchronization therapy and defibrillation (*CRT‑D*) system comprising a Micra leadless pacemaker in the right ventricle (RV), a WiSE-CRT system, and an Emblem subcutaneous implantable cardioverter defibrillator (*S‑ICD*). **a** ECG during RV pacing from the Micra leadless pacemaker (QRS duration 210 ms). **b** ECG during biventricular pacing from the Micra leadless pacemaker and WiSE-CRT system (QRS duration 158 ms). **c** Postero-anterior and **d** lateral chest X‑ray images demonstrating the components of the leadless CRT‑D system. (Reproduced with permission from Sidhu et al. 2020 [37])

The majority of WiSE-CRT systems are implanted in patients with existing conventional lead-based pacemakers or implantable ICDs. However, completely leadless CRT can be achieved with the WiSE-CRT system in combination with a leadless pacemaker, and feasibility has been demonstrated in a small multicenter series of eight patients [36]. The addition of a subcutaneous ICD has also been demonstrated, to achieve a completely leadless CRT–defibrillation system (Fig. 2; [37]). The combination of the WiSE-CRT system with a leadless RV pacemaker can only achieve ventricular resynchronization, and therefore is only an option for patients in chronic atrial fibrillation. However, the use of the Micra-AV (Medtronic, Fridely, MN, USA) could potentially make additional atrioventricular resynchronization possible and thus extend the utility of this combination to patients in sinus rhythm. Entirely leadless pacing systems are an attractive option for patients with recurrent lead complications or vascular access issues, such as hemodialysis patients, and are likely to be an area of increased interest in the future.

### Leadless conduction system pacing

His bundle pacing and left bundle branch area pacing (LBBAP) are novel therapies that can engage the intrinsic His–Purkinje system to achieve cardiac resynchronization, and may be viable alternatives to endocardial LV pacing for patients in whom conventional CRT failed [38]. While both therapies are delivered using conventional lead-based technology via the right heart, temporary LBBAP from the left ventricular aspect of the septum has been demonstrated, with superior electrical resynchronization compared to conventional CRT [39]. Although the conventional target for the WiSE-CRT endocardial electrode has been the LV lateral wall, successful implantation in the LV septum has been reported, with subsequent delivery of leadless LBBAP [40]. Further study of the efficacy and safety of leadless LBBAP using the WiSE-CRT system is required, including how the required simultaneous pacing of the right ventricle affects cardiac resynchronization.

## Conclusion

Endocardial LV pacing can achieve cardiac resynchronization and may offer distinct advantages over conventional CRT, including a wider choice of pacing sites and potentially superior electrical resynchronization and hemodynamic response. The delivery of endocardial LV pacing using conventional lead-based technologies has been demonstrated in observational studies, but is limited by the risk of thromboembolic complications and the need for long-term anticoagulation. These risks may be mitigated by wireless endocardial pacing via the WiSE-CRT system, and the current SOLVE-CRT trial will provide important information on the safety and efficacy of this system. The combination of the WiSE-CRT system with leadless pacemakers to deliver entirely leadless CRT, and the implantation of the endocardial electrode in the septum to achieve leadless left bundle branch area pacing, are other novel areas for potential future research.

## Declarations

For this article no studies with human participants or animals were performed by any of the authors. All studies performed were in accordance with the ethical standards indicated in each case.
